# iTCep: a deep learning framework for identification of T cell epitopes by harnessing fusion features

**DOI:** 10.3389/fgene.2023.1141535

**Published:** 2023-05-09

**Authors:** Yu Zhang, Xingxing Jian, Linfeng Xu, Jingjing Zhao, Manman Lu, Yong Lin, Lu Xie

**Affiliations:** ^1^ School of Health Science and Engineering, University of Shanghai for Science and Technology, Shanghai, China; ^2^ Shanghai-MOST Key Laboratory of Health and Disease Genomics, Institute of Genome and Bioinformatics, Shanghai Institute for Biomedical and Pharmaceutical Technologies, Shanghai, China; ^3^ Bioinformatics Center, National Clinical Research Centre for Geriatric Disorders, Department of Geriatrics, Xiangya Hospital, Central South University, Changsha, Hunan, China; ^4^ Ministry of Education Key Laboratory for Biodiversity Science and Ecological Engineering, Institute of Bio-Diversity Science, School of Life Sciences, Fudan University, Shanghai, China

**Keywords:** iTCep, T cell epitopes, peptide-TCR interaction, immunotherapy, deep learning modeling

## Abstract

Neoantigens recognized by cytotoxic T cells are effective targets for tumor-specific immune responses for personalized cancer immunotherapy. Quite a few neoantigen identification pipelines and computational strategies have been developed to improve the accuracy of the peptide selection process. However, these methods mainly consider the neoantigen end and ignore the interaction between peptide-TCR and the preference of each residue in TCRs, resulting in the filtered peptides often fail to truly elicit an immune response. Here, we propose a novel encoding approach for peptide-TCR representation. Subsequently, a deep learning framework, namely iTCep, was developed to predict the interactions between peptides and TCRs using fusion features derived from a feature-level fusion strategy. The iTCep achieved high predictive performance with AUC up to 0.96 on the testing dataset and above 0.86 on independent datasets, presenting better prediction performance compared with other predictors. Our results provided strong evidence that model iTCep can be a reliable and robust method for predicting TCR binding specificities of given antigen peptides. One can access the iTCep through a user-friendly web server at http://biostatistics.online/iTCep/, which supports prediction modes of peptide-TCR pairs and peptide-only. A stand-alone software program for T cell epitope prediction is also available for convenient installing at https://github.com/kbvstmd/iTCep/.

## 1 Introduction

Effective targeted immunotherapy requires accurate prediction of which tumor-specific epitopes are most likely to trigger an immune response from T cells. Neoantigens, also called tumor-specific antigens (TSA), are mutated peptides derived from the expression of mutated genes in tumor cells and presented on the tumor cell surface by major histocompatibility complex (MHC) and subsequently trigger a neoantigen-specific T cell response to destroy tumors. As a key role to initiate an immune response, T cell activation occurs only when the T cell receptors (TCR) recognize peptide-MHC (pMHC) complexes (Szeto et al., 2020; Schaap-Johansen et al., 2021). The TCR complementary determing region 3 (CDR3) that derived from quasi-random mutations of V(D)J recombination is considered to be the main driver for recognizing the highly polymorphic MHC and large repertoire of peptides (Chiffelle et al., 2020). The random rearrangement of TCR gene fragments could generate more than $10^{15}$ T cell clonetypes, with each expressing a particular TCR and thus contributes to specific epitopes recognition. Tetramer analysis and tetramer-associated T cell receptor sequencing can be used to verify the binding of pMHC and TCR pairs (Altman et al., 1996; Zhang et al., 2018). However, these experimental methods are time-consuming and generally technic-challenging to perform. Consequently, there remains an urgent need for methods that can accurately characterize antigens and TCR interactions, which will contribute to clinical, therapeutic, and pharmaceutical applications in the design of tumor immunotherapies.

The development of high-throughput TCR sequencing techniques has accelerated the availability of epitope-specific TCR sequences. With the emergence of public databases containing large-scale experimentally validated epitopes such as McPAS-TCR, VDJdb, and IEDB (Nili et al., 2017; Swapnil et al., 2018; Bagaev Dmitry et al., 2020), an increasing number of computational approaches for TCR-epitope binding have become available. In the past several years, some tools, such as NetTCR (Montemurro et al., 2021), TCRex (Gielis et al., 2019), ERGO (Springer et al., 2020), imRex (Moris et al., 2021), have witnessed the possibility and feasibility of generating a model to identify the specificity of TCRs binding to an epitope. Theoretically, 9-mer-restricted models like NetTCR cannot be applied to an out-of-length epitope, and epitope-specific models like TCRex cannot be applied to an unknown epitope. To tackle these issues, novel methods should be designed to expand the application scope of T cell epitope identification models while improving their accuracy and generalization.

Recent advances in deep learning for genomics (Liu et al., 2020), proteomics (Meyer, 2021), protein structure prediction (Pakhrin et al., 2021), immunotherapy (Tran et al., 2020), etc. have highlighted its effective application in the field of biomedicine (Sapoval et al., 2022; Tran et al., 2022). Compared with traditional machine learning, deep learning has unique advantages mainly to automatically learn complex multi-level data representation and superior performance. Previous work in our group has addressed the candidate neoantigen prediction problem from an immunogenicity prediction angle. We presented a model named DeepCNN-Ineo, which considered information about binding affinity of peptide-MHC and the immunogenicity of neoantigen peptide-side to increase the reliability of prediction (Lu et al., 2022). Unfortunately, only simply knowing the immunogenicity of the candidate peptide alone is not sufficient to accurately infer TCR-specific epitopes.

In this work, we proposed iTCep, a deep learning framework to predict TCR-epitope recognition that was inspired by intermediate fusion in multimodal fusion strategy (Boulahia et al., 2021). Firstly, a novel feature representation method was presented to convert sequences into interaction maps, which calculates the positional probabilities on the amino acid level. Next, two different interaction maps together as a fusion feature, which concatenates the resulting feature vectors from different layers of neural networks for subsequent classification. In addition, McPAS-TCR and dbPepNeo2.0 data were collected to independently evaluate the generalization of the proposed model. We focused on the interaction between peptides presented by MHC class I molecules and the CDR3 variable regions of TCR β-chain, which are directly linked with anchor residues of antigens and thus being a key role in the prediction of T cell recognition specificity (Lu et al., 2021).

## 2 Materials and methods

### 2.1 Data collection

To construct an optimum positive dataset for model training and testing, we collected experimentally verified immunogenic epitopes and their recognizing TCRs from three publicly available T cell epitope databases, i.e. McPAS-TCR (database release 5 August 2021), VDJdb (database release 30 March 2022), and IEDB (Nili et al., 2017; Swapnil et al., 2018; Bagaev Dmitry et al., 2020). Epitopes recognized by CD8+ T cells from the McPAS-TCR were selected, covering three key issues (Human, Cancer, Neoantigen). Moreover, to further evaluate the performance of peptide-TCR binding predictors, we collected experimentally validated peptides and CDR3 sequences from dbPepNeo2.0 (Lu et al., 2022), a database for human tumor neoantigen peptides previously developed by our group.

To create negative dataset, we collected the TCR beta chain sequences originating from healthy individuals in TCRdb, the public comprehensive database for T cell receptor sequences (Si-Yi et al., 2021). In this research, we used samples from project PRJNA390125 (sample IDs: SRR5676643, SRR5676656, SRR5676661) to obtain TCR repertoire of healthy controls. In general, negatives are defined as pairs of peptides and TCRs that do not interact. We created negative data by using random pairings of immunogenic peptides from the positive dataset and sequences from healthy individuals, assuming that they did not generate a tumour-specific immune response as they are less likely to have interactions.

### 2.2 Data processing

From the aforementioned datasets, we gathered a total of 16,746 pairs of TCR CDR3 and their recognized epitopes, which were then processed in accordance with the detailed procedures outlined below. 1) For the VDJdb dataset, TCR-epitope combinations with a confidence score higher than zero were retained. 2) Samples with missing epitopes or CDR3 sequences were eliminated, as were those with improper sequence formats, such as spaces and unknown amino acids. 3) We discarded duplicated peptide-TCR CDR3 sequences resulted from ignoring the information such as the CDR1, CDR2 regions, V/D/J genes, and HLA molecules. 4) Given that mass spectrometry analysis of MHC class I-presented peptides has revealed that highly conserved, short AAs are the most abundant peptides, we adopted peptides with 8–11 AAs and TCR CDR3 with 8–21 AAs, respectively.

**FIGURE 1:**
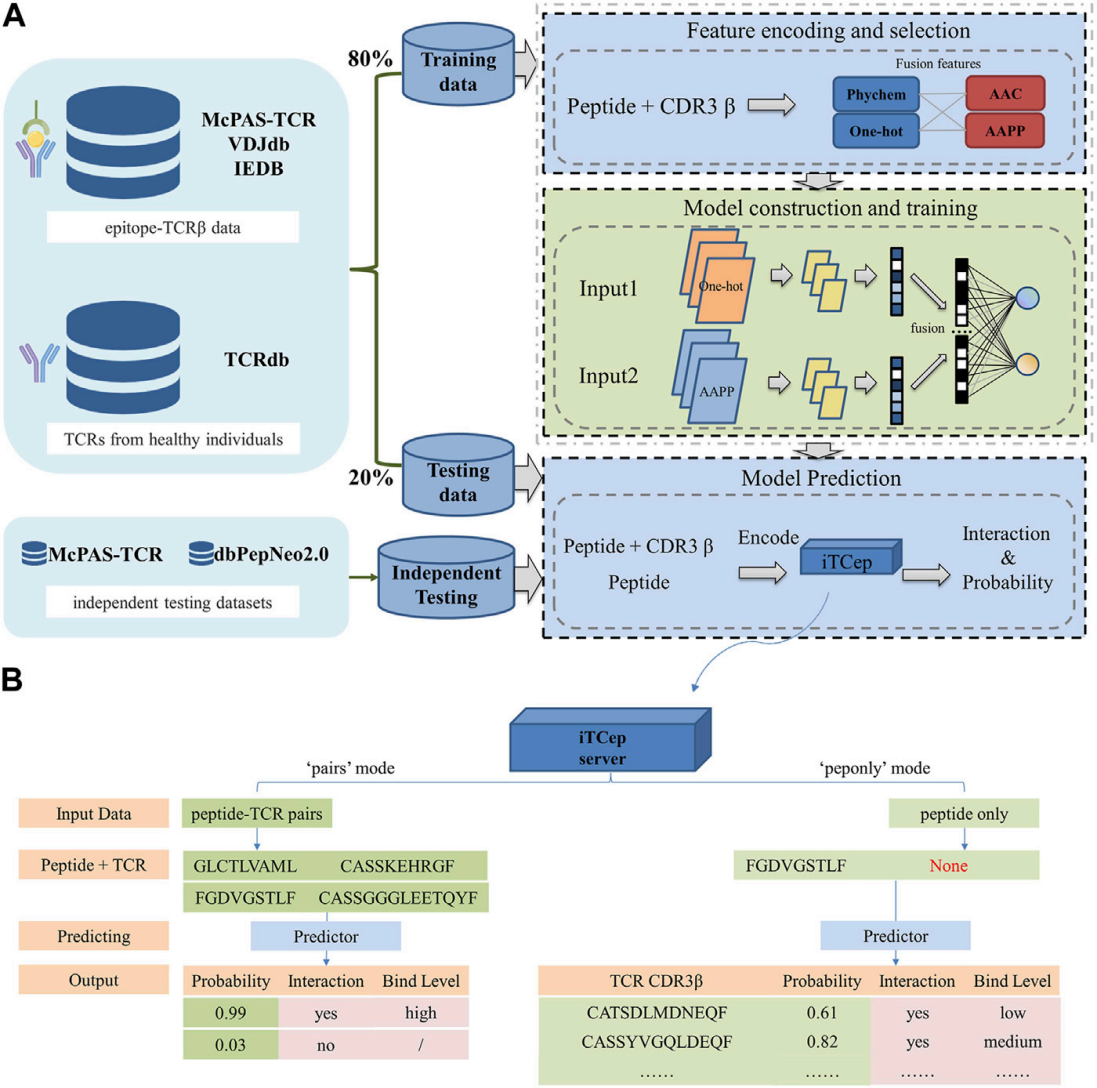
Workflow of the proposed predictor iTCep. **(A)** The deep learning-based framework iTCep is graphically illustrated with several key modules: data collection, feature encoding and selection, model construction and training, model prediction. **(B)** Function of iTCep’s prediction. The iTCep can predict the interaction of peptides and TCRs according to the output classification values of the model selected by the user.

After processing, the curated positive dataset was whittled down to 10,759 pairs of TCR-epitope bindings, covering a total of 329 unique epitopes (Supplementary Figure S1). Consequently, the positive dataset and the randomly sampled negative dataset were combined as the final dataset for training and testing (Figure 1A). In addition, the McPAS-TCR dataset was further partitioned into a peptide-shared subset (McPAS-shared) containing epitopes that already present in the training data, and a peptide-unique subset (McPAS-unique) containing epitopes that ever unknown in the training data. Two independent testing datasets were used for model performance evaluation: McPAS-unique containing 243 TCR-epitope pairs covering 18 unique epitopes, and another dataset dbPepNeo2.0, containing 332 TCR-epitope pairs spanning 44 unique epitopes. Details about all the distribution of datasets used in this study are shown in Table 1. The overall dataset for model construction can be downloaded from the web http://biostatistics.online/iTCep/#/download.

**TABLE 1:** Sources and distribution of sample numbers in the overall dataset.

Source	McPAS-TCR (shared)	VDJdb	IEDB	TCRdb (health)	dbPepNeo 2.0	McPAS-TCR (unique)
Positive	6,899	3,678	182	—	332	243
Negative	—	—	—	10,759	—	—

### 2.3 Feature encoding for deep learning framework

#### 2.3.1 One-hot encoding

One-hot encoding is the process of converting a category variable into a format that can be easily utilized by machine learning algorithms. Therefore, it is a simple and straightforward solution that transforms the amino acid sequences into binary features in most cases. In this work, TCRβ CDR3 and peptide sequence pairs of varying lengths were zero-padded, resulting in matrixes with 21 rows × 20 columns and 11 rows × 20 columns respectively.

#### 2.3.2 Phychem encoding

In order to describe the biological properties of amino acids in detail, we adopted phychem encoding method using physicochemical properties, such as polarity, hydrophobicity, charge, etc. They were gathered from Protscale (Walker, 2005) (Supplementary Table S1) to encode the CDR3 and peptide sequences. This feature is based on the biological principles that the different chemical properties derived from the different amino acids can affect their interactions with other molecules. These generated numerical feature matrixes with 21 dimensions were finally standardized because of the significant discrepancies in values. Consequently, all data were assigned to a normal distribution with a mean of 0 and a variance of 1.

#### 2.3.3 AAC and AAPP encoding

Considering the molecular interaction between TCR and peptide sequences, we utilized two encoding strategies to represent the distribution of amino acids. The natural structure and functioning of a protein in a given environment are notably influenced by the amino acid composition (AAC). It is a typical attribute used to estimate the probability of amino acids occurring in the flanking region of PTM sites (Kao et al., 2020). The amino acid composition is measured as the proportion of amino acids in a sequence standardized by the total number of residues (Gromiha, 2010). This implies that it represents the occurrence frequency of each amino acid in the TCR or peptide sequence. It can be defined as:

$$AAC\left(i\right)=\frac{{count}_{i}}{N_{s}}$$

where i represents the 20 amino acid residues; ${count}_{i}$ is the number of each sort of residue in a peptide or CDR3 sequence, and $N_{s}$ is the total number of residues in peptide.

In order to further investigate the amino acid composition from the perspective of interactions between epitopes and their recognizing TCR CDR3 sequences, we devised an approach called Amino Acid Position Preference (AAPP). It calculates the probability of the amino acid position in epitope-specific TCR repertoire. This feature is based on the biological principle that different positions in a sequence can have different effects on TCR-epitope interactions. It can be defined as:

$$AAPP\left(p_{i}\right)=\frac{count\left(x,i\right)}{N_{t}},x=2,3,\ldots ,21$$

where i represents the 20 amino acid residues; $p_{i}$ represents a unique peptide in positive dataset; x is the position of amino acid in CDR3 sequence, ranging from the second to the 21st because of the same start cysteine; $count\left(x,i\right)$ is the number of each residue in CDR3 sequence at position x ; $N_{t}$ is the total number of TCRs that specifically recognize the unique epitopes.

### 2.4 Deep learning model construction

We constructed a dual-input deep learning architecture using fusion features for peptide-CDR3 interaction prediction. Firstly, due to the inconsistency in the feature dimension, feature maps computed by AAPP were transposed, where the row correspond to the positions and the col to amino acids. For methods other than AAPP, matrixes obtained from peptide and TCR respectively were concatenated for capturing relationship from feature representation of sequences. Subsequently, we divided the encoding methods stated above into two groups: one for single amino acid encoding, i.e., one-hot and physicochemical property, and another for amino acid distribution, i.e., AAC and AAPP. Methods from both groups were integrated to create four fusion features for subsequent model training: onehot-AAC, onehot-AAPP, phychem-AAC and phychem-AAPP. Next, convolutional neural networks (CNN) were applied to construct the final predictor in our experiments, which can effectively extract deep features owing to their high self-learning abilities (Dong et al., 2022).

### 2.5 Model training and performance evaluation

The Adagrad optimizer with a learning rate of 0.01 was used to reduce the losses through calculating the gradients of all parameters. Mean Squared Error (MSE) loss was used as the objective function to measure the prediction of peptide-CDR3 pairs. Models were trained for 60 epochs with a batch size of 20, which proved adequate by observing the training loss curves.

The results of models were verified with 5-fold cross-validation (CV) on same CV sessions, which were repeated four times by setting different random seeds to draw a stable conclusion. With this strategy, the receiver operating characteristic (ROC) curve and the area under the ROC curve (AUC) value were obtained to evaluate classifiers intuitively. Furthermore, we applied the precision, accuracy, recall, f1-score and matthews correlation coefficient (MCC), the metrics commonly used in classification tasks, to evaluate the performance of predictors. The final model was determined by model in last epochs in the last CV repeat.

We implemented and trained our models using TensorFlow 2.4.0 (Abadi et al., 2016) backend, Keras 2.6.0 (https://github.com/keras-team/keras) and the Python (3.7.6) packages Biopython 1.76 (Cock et al., 2009), NumPy 1.19.5 (Walt et al., 2011), pandas 0.25.3 (McKinney, 2010), Scikit-learn 0.24.2 (Pedregosa et al., 2011) and SciPy 1.4.1 (Virtanen et al., 2020).

### 2.6 Prediction on unknown epitopes

Since the encoding method AAPP is based on amino acid position distribution of seen epitopes and their corresponding TCRs, the model will not be able to make a precise judgement based on the knowledge learned from training while unknown peptides are encountered. Considering this issue, we developed a novel strategy to encode the peptide-TCR pairs that are not appeared in training dataset. The peptide with the highest similarity to the target was found by applying the minimum edit distance (MED) algorithm, also known as Levenshtein distance, which refers to the smallest number of operands required to transform one sequence to another (Levenshtein, 1965; Shuwandy et al., 2020). Only three editing operations including insertion, deletion and substitution on single-character can be performed. The MED of sequence A and B can be described as:

$${lev}_{A,B}\left(i,j\right)=\left\{\begin{array}{l} \max\left(i,j\right)if\min\left(i,j\right)=0,\\ \min\left\{\begin{array}{c} {lev}_{A,B}\left(i-1,j\right)+1\\ {lev}_{A,B}\left(i,j-1\right)+1\\ {lev}_{A,B}\left(i-1,j-1\right)+1_{\left(A_{i}\neq B_{i}\right)} \end{array}\right.\;otherwise \end{array}\right.$$

where ${lev}_{A,B}\left(i,j\right)$ indicates the distance between the first i characters of A and the first j characters of B.

## 3 Results

### 3.1 Overview of the model architecture for predicting peptide-TCR binding

**FIGURE 2:**
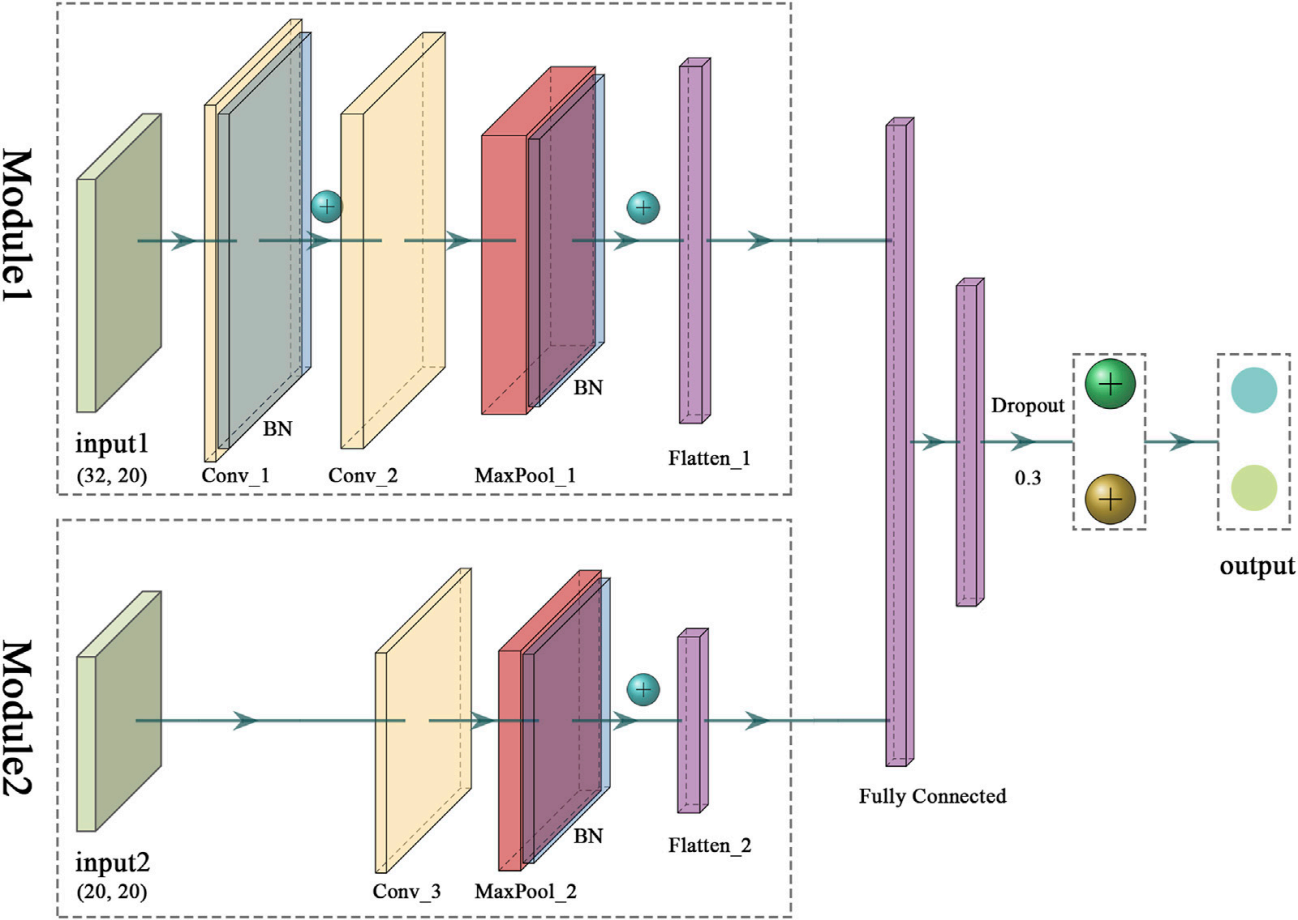
The architecture of iTCep. The peptide-TCR interaction feature maps generated by two encoding methods were provided to the model in separate layers and then being concatenated to a fully connected layer. Convolutional neural networks with two single-feature inputs are represented in Modules 1 and 2, respectively. BN, Batch Normalization Layer.

The architecture with two modules was adopted to capture underlying differences across sequence distribution caused by VDJ recombination mechanisms of TCRs and antigen generation and processing (Figure 2). More specifically, two feature maps are fed independently into the input layers of modules, which accepts 640 and 400 variables, respectively. Following that, for Module1, a 2D convolutional layer with 16 filters with kernel size of 3 × 2 and another with 32 filters with kernel size of 6 × 4 are adopted, succeeded by a max pooling layer with pooling kernel size of 2 and stride of 1, which is used to reduce the feature dimension and avoid overfitting. For Module2, a 2D convolutional layer with 16 filters with kernel size of 1 × 2 and a max pooling layer with pooling kernel size of 2 and stride of 1 are positioned after the input layer. Batch normalization layers are added after the first convolutional layer of Module 1 and every max pooling layer. Each module ends with a fully connected layer that is used to create connections between different features and combine them into a single layer, followed by two fully connected hidden layers with 256 and 128 variables, both using L2 regularization with penalty of 0.01.

In addition, except for the output neuron, all neurons used rectified linear unit (ReLU) as the activation function while training using backpropagation. The output layer has two variables and the Softmax activation function can be utilized to obtain the output value of the classification. A dropout layer with the probability of 0.3 for connections between the last hidden layer and output layer is added to temporarily remove units of neural network.

### 3.2 AAPP encoding results in improved accuracy on the prediction of peptide-TCR interactions

**FIGURE 3:**
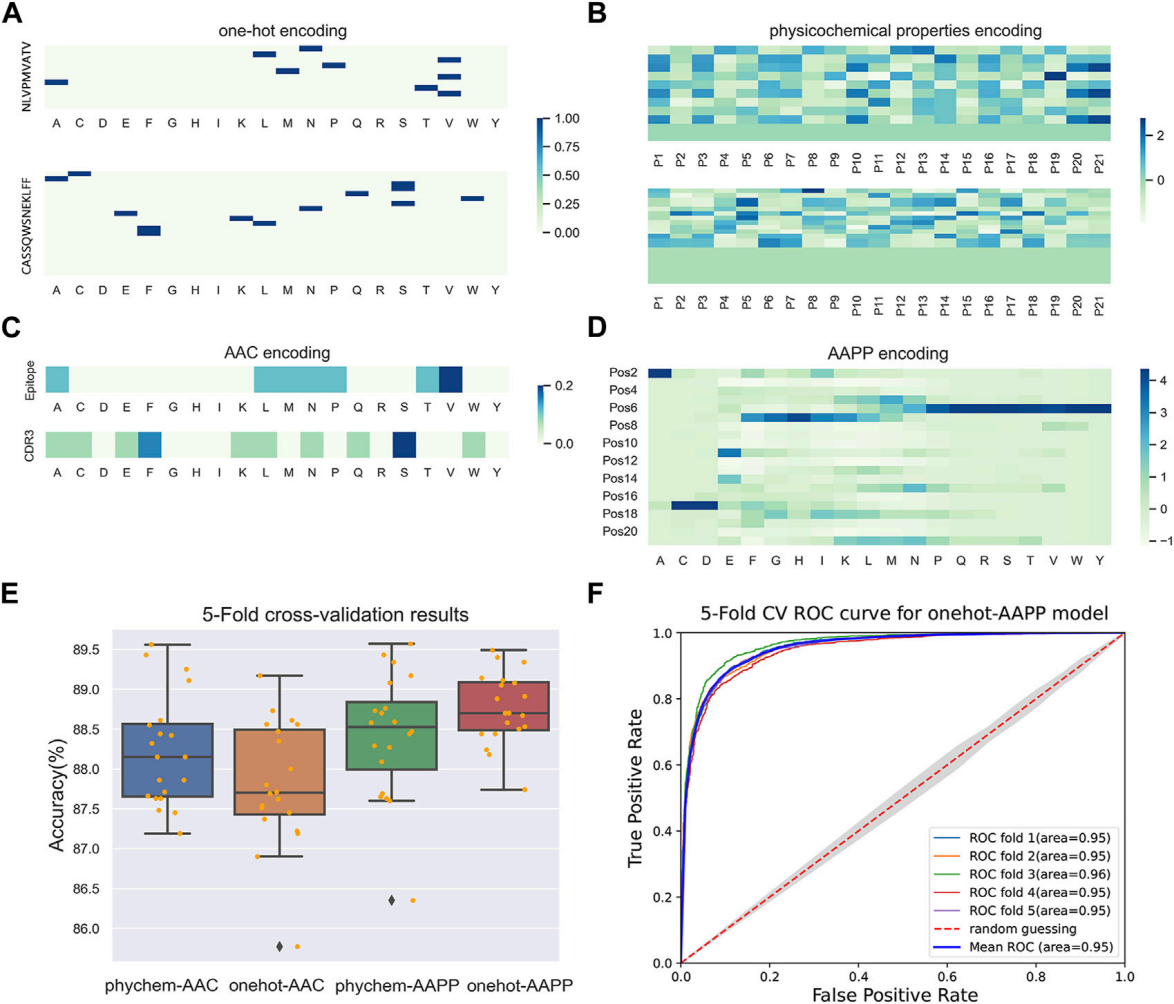
Model training for predicting interaction between peptide and TCR. **(A–D)** The interaction feature map created by **(A)** one-hot encoding, **(B)** physicochemical properties encoding, **(C)** AAC encoding and **(D)** AAPP encoding for epitope (NLVPMVATV) and TCR (CASSQWSNEKLFF) interaction representation. **(E)** 5-fold CV results of model training based on different fusion features. **(F)** Validation receiver operating characteristic (ROC) for the iTCep with shuffled samples using 5-fold CV on training dataset. The average level of AUC is depicted by the dark blue ROC curve.

To compare different feature fusion strategies and choose one that could be conducive to build a model with high prediction accuracy, the peptide-CDR3 pair sequences were padded to the maximum length of 32 and were converted into feature matrixes with variant dimensions using onehot-AAC, onehot-AAPP, phychem-AAC and phychem-AAPP, respectively. Subsequently, we trained the deep learning model built using previously mentioned architecture and performed parameter tuning with tuner to capture the optimal hyper-parameters. Each interaction feature map, as a separate input to be concatenated in model construction, can be observed as an image. The feature dimension of the input layer is determined by the input features. An example of epitope (NLVPMVATV) and TCR (CASSQWSNEKLFF) is given in Figures 3A–D. The AAPP feature map shed light on the relationship between the epitope-specific TCRs as a whole, in contrast to one-hot feature maps, which arrange the sequence of the epitope and properties on the axes of a two-dimensional matrix. Such two types of features complement one another and then support the representation of peptide-TCR interactions.

The 5-fold cross-validation with four times independently repeat was applied to infer whether fusion feature could benefit model performance improvement and to investigate the random influence of data division. The results showed that each model achieved high accuracy, with a minimum value of 85.56% (Figure 3E), indicating that the proposed models showed reliable predictions. Therein, it is noteworthy that AAPP encoding based fusion features had a greater positive impact on the prediction of peptide-TCR interactions than that based on AAC. Models built on onehot-AAPP encoding methods achieved the highest accuracy, with an average value of 88.76% (Supplementary Table S2), which may attribute to the fact that the sparse matrix generated by one-hot is more suitable for training convolutional neural networks. Similarly, the phychem-AAPP based models also achieved good predictions with a slightly poorer performance on validation data during 5-fold CVs, demonstrating that the novel encoding method could accomplish improvement on the representation of peptide-TCR interactions.

Furthermore, we reserved models in the last epoch of last cross-validation repeat during the 5-fold cross-validation process and compared their performance on the testing dataset. We noticed that the model of onehot-AAPP, namely iTCep, outperformed other predictors in terms of most metrics including accuracy, recall, F1-score and MCC (Table 2). The average area under the receiver operating characteristic (AUROC) over the iTCep is up to 0.95, suggesting that the deep learning networks with multiple layers were capable of recognizing the variations in sequence between peptide-TCR pairs (Figure 3F).

**TABLE 2:** Comparative performance results of models constructed based on different fusion features on the testing dataset.

Metrics	Phychem-AAC	Onehot-AAC	Phychem-AAPP	Onehot-AAPP
Precision	0.889	0.852	**0.912**	0.877
Accuracy	0.885	0.875	0.888	**0.893**
Recall	0.881	0.908	0.858	**0.914**
F1-score	0.885	0.878	0.884	**0.895**
MCC	0.770	0.751	0.777	**0.786**

The bold values represent the best performance metrics of the models.

### 3.3 iTCep performs better than conventional machine learning classifiers

To emphasize superior performance of the proposed architecture, we compared several classifiers utilized classical machine learning algorithms including support vector machines (SVM), decision tree (DCT), random forest (RF) and AdaBoost. Features that standardized to a normal distribution described in feature encoding for deep learning section were used to train these classifiers. Additionally, the testing dataset and the McPAS-unique dataset (Table 1) were both recruited for model performance validation.

The detailed performance results of these conventional models are shown in Supplementary Table S3. Therein, features most applicable to each algorithm were explored and models with the best performance on testing data and independent testing data were chosen for comparative analysis, as depicted in Table 3. Interestingly, we found that the optimal features applied by different classifiers varied considerably on both testing sets, while iTCep kept consistently with the highest performance. Random forest classifiers had the highest prediction accuracy on the testing set, but showed lower AUCs than iTCep. For independent testing I, it can be clearly observed from the tables that models based on deep learning outperformed other classification methods with improved AUC. This implied that deep learning based on fusion features performed more effective and robust than other classification approaches in terms of performance improvement for the prediction of peptide-TCR CDR3β interactions.

**TABLE 3:** Comparison of the performance on different classifiers for predicting peptide-TCRβ interaction based on testing dataset and independent testing dataset I.

Classifier	Feature	Testing dataset		Feature	Independent testing data I	
		ACC (%)	AUC		ACC (%)	AUC
iTCep	Onehot, AAPP	89.29	**0.955**	Onehot, AAPP	**85.80**	**0.909**
SVM	Onehot, AAC	89.20	0.892	Phychem, AAPP	84.16	0.842
DCT	Onehot, AAPP	87.36	0.874	Onehot, AAPP	73.87	0.739
RF	Phychem, AAC	**90.45**	0.905	Onehot, AAPP	85.19	0.852
Adaboost	Phychem, AAPP	88.06	0.881	Phychem, AAPP	83.33	0.833

Abbreviations: ACC: accuracy; AUC: area under the receiver operating characteristic curve. The bold values represent the best performance metrics of the models.

**FIGURE 4:**
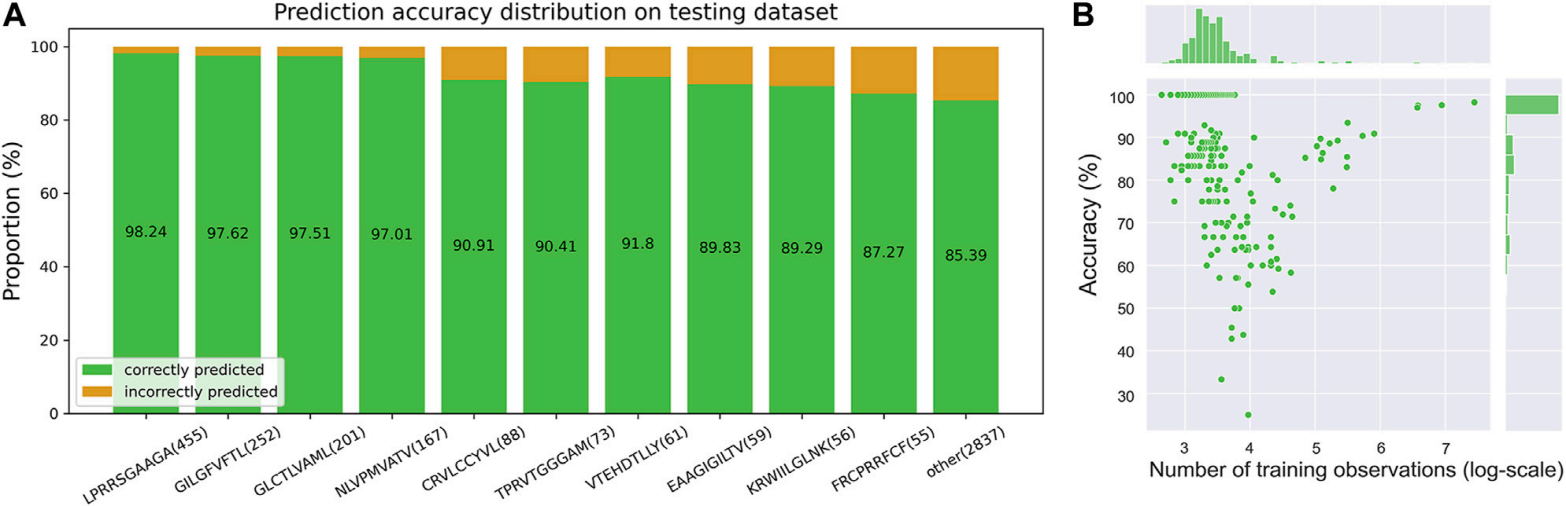
iTCep for peptide-TCR prediction. **(A)** Prediction performance of the iTCep classifier for TCR cross-reactivity on the peptide-TCR pair dataset. Peptides and their numbers in test samples are represented by the horizontal coordinates, while the proportion of correctly (green) or erroneously (orange) predicted peptide-TCR pairs is represented by the vertical coordinates. The ACC values of the iTCep used to predict the interaction between peptide and TCR are displayed in green bars. **(B)** Scatterplot of accuracy on testing data and training sample size for each epitope.

Finally, in order to explore the predictive power of the iTCep for TCR cross-reactivity, we applied the model to the testing dataset, in which peptide-TCR pairs appeared in training dataset were filtered out. Specifically, the top 20 peptides (Supplementary Figure S2) with the most abundant TCRs out of the overall epitopes were counted and the top ten were further filtered to calculate the prediction accuracy of the iTCep. From the prediction accuracy distribution on testing dataset depicted in Figure 4A, it can be perceptible that training samples with a larger number of TCRs resulted in higher prediction performance. However, this conclusion does not seem to hold true for the rest of epitopes with small-scale of cognate T cell receptors, since no correlation was found between the number of training samples and the accuracy of an epitope (Figure 4B). Consequently, we speculated that the overall performance of iTCep was mostly influenced by abundant epitopes, which explains the misleading results of a general model in certain situations.

### 3.4 iTCep obtains equivalent performance with state-of-art peptide-TCR binding approaches

In recent years, several new tools have been published to predict the binding of peptides to TCRs. The models ERGO-AE and ERGO-LSTM, which were trained on autoencoder (AE) and long short-term memory (LSTM), respectively, applied Natural Language Processing (NLP) to create TCR-peptide binding predictors (Springer et al., 2020). In both models, a multilayer perceptron (MLP) with one hidden layer was employed to obtain the binding probability value. Moris et al. (2021) presented a novel interaction map recognition (imRex) method that based on the pairwise combination of physicochemical properties. This approach can be applied to predict previously unseen epitopes in training data. The DLpTCR model was proposed by Xu et al. (2021), using ensemble deep learning consisted of three base classifiers for single/paired chain(s) of TCR and peptide interaction prediction. TetTCR-seq and VDJdb datasets were used to train this model, and data from both VDJdb and IEDB were used to perform validation. These methods performed well in predicting the interaction between peptide and TCR β chain. Therefore, we compared the performance of iTCep with these predictors to shed light on the superiority of our proposed model.

**FIGURE 5:**
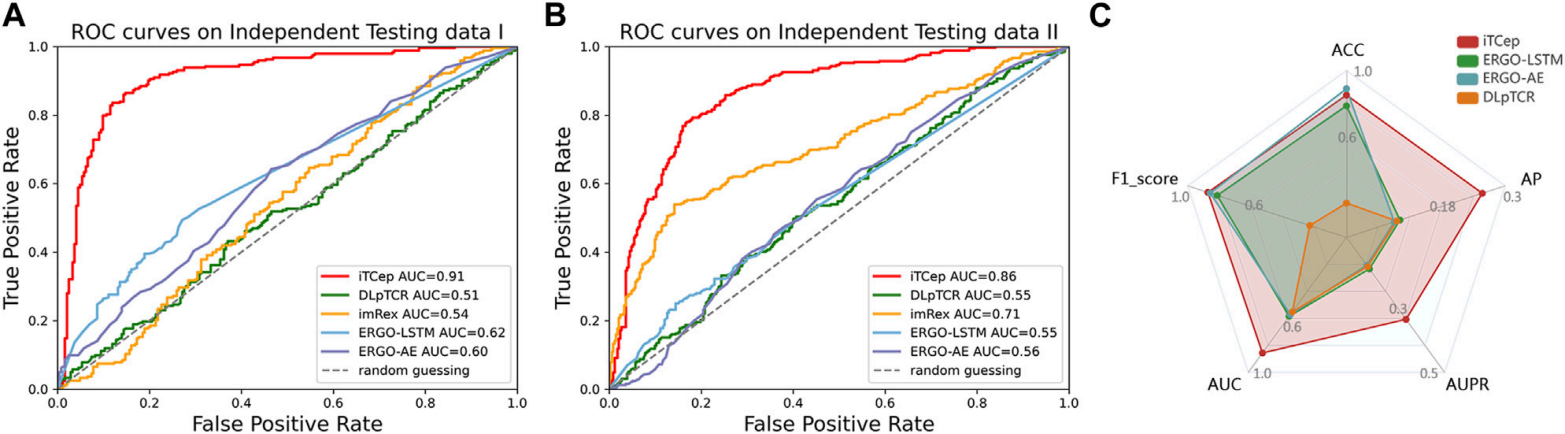
Comparison of model prediction performance between iTCep and the published methods on independent testing datasets. **(A)** ROC curves and AUC values for predictors on the McPAS-unique dataset. **(B)** ROC curves and AUC values for predictors on the dbPepNeo2.0 dataset. **(C)** Comprehensive performance comparison of the four predictors on the unbalanced dataset.

McPAS-unique dataset was adopted to valid the predictive performance on the interactions of TCR-epitope covering unseen peptides or novel TCRs. According to the results of ROC curves (Figure 5A), it can be easily observed that the iTCep achieved an AUC of 0.91 on McPAS-unique data, while the DLpTCR achieved the lowest AUC of 0.51. These results indicated that the iTCep model attained the same level of performance on unique epitopes of McPAS-TCR as it did on shared epitopes during cross-validation. Moreover, when applying the predictors on high-confidence neoantigens in dbPepNeo2.0 dataset, the iTCep still demonstrated a better performance (AUC = 0.86) in contrast to other previous mentioned models (Figure 5B). To validate the predictive performance of the predictors on real data distribution, a larger scale of independent dataset was created, with a 10:1 ratio of negative and positive pairs. According to the evaluation metrics of iTCep, it demonstrates superior overall performance on imbalanced data compared to other models (Figure 5C). In conclusion, iTCep could obtain equivalent performance with state-of-art peptide-TCR binding approaches according to the performance comparisons on independent datasets, outperforming other current tools of similar purposes.

### 3.5 The iTCep server for T cell epitope prediction

A web application named iTCep was developed from the pretrained classifiers to predict the interactions between peptides and TCR beta chain sequences. The iTCep webserver provides two predicting functionalities, one to predict the interactions between the given multiple peptide-TCR pairs and the other to obtain the TCRs that could recognize the input peptides in accord with ranked predictive values (Figure 1B). The latter function was added in order to bring some informative significance to the prediction of TCRs for users who have only peptides and no TCR CDR3 sequences.

With the ability to input sequences or upload files, users can submit their own peptide-TCRβ pairs for prediction. The predicted results are presented in five columns, including peptide, TCR CDR3, probability, interaction, and binding level. Users can choose to receive the result file via email or access it directly on the web page. Binding affinity between peptide-TCR pairs is classified into three levels based on customized thresholds. Peptide-TCR pairs with a probability greater than 0.5 are considered to be truly combinations. Among them, probability scores less than 0.8 indicate low level, scores greater than 0.95 indicate high level, and scores between 0.8 and 0.95 indicate medium level of binding. These definitions are important for accurately assessing the strength of the predicted interactions between peptides and TCRs.

In summary, the iTCep webserver enables researchers to identify peptides with high immunogenicity, allowing further screening of neoantigens to maximize the benefit of immunotherapy to patients. This web tool was built using the Vue.js web framework while the back-end was implemented by Flask 2.0.2 (24), it is accessible at http://biostatistics.online/iTCep/.

## 4 Discussion

Peptide binding to MHC molecules has been the main focus of many epitope predictions. However, not all peptides presented by MHC molecules are immunogenic. TCR must interact with the pMHC complex to trigger an immune response. However, the prediction pipeline based on multiple omics data will generate tens of thousands of candidate epitopes, and it is difficult to filter all possibilities through experimental verification. Machine learning especially deep learning algorithms have been developed to explore the interaction between TCR and pMHC to further narrow down the range of positive neoantigens in MHC presenting peptides.

In this study, a novel feature AAPP was introduced and applied to represent peptide-TCR interactions. Then we compared prevailing encoding metrics and utilized fusion features that served as inputs of deep learning models, which were trained with multi-layer convolutional neural networks using cross-validation. Given the additional information provided by the physicochemical properties, it would be expected to contribute more to the accuracy of the predictions. However, the results seem to disprove this hypothesis, as the overall performance in the CV test remains poor. Instead, the fact that the fusion of one-hot and AAPP may help avoid overfitting and thus improve the generalization performance. In general, the individual performance of an epitope is assumed to have a positive correlation with the diversity of its TCR repertoire, whereas no discernible patterns were found in the final accuracy and the number of training examples. Furthermore, our approach was validated on several independent datasets to confirm the improved performance from different research perspectives. We found that iTCep achieved surprising results with prediction accuracy of 85.80% and AUC of 0.909 on the McPAS-TCR dataset, indicating that the iTCep could capture differences among features of shared and unique epitopes. Comparatively, other predictors reached lower prediction performance than our proposed model, in particular imTCR and DLpTCR, which are also based on CNN architectures. Similar conclusions can be drawn when the dbPepNeo2.0 dataset was applied. We also assessed these current models in an unbalanced task in addition to their performances on balanced datasets. The comparison of iTCep’s AUCs and ACCs with state-of-art models proved its high sensitivity and better generalization capabilities in identifying true epitopes.

Since our model was primarily trained on tumor antigens, we hypothesized that our model may not perform as well on predicting binding affinities for epitopes outside this range. As a matter of fact, iTCep performed poorly in predicting TCR-epitope pairs derived from COVID-19 data in DLpTCR’s reference set. To investigate the scalability of iTCep, we applied transfer learning to iTCep using COVID-19 data and compared our model’s performance with that of DLpTCR (Supplementary Figure S3). The results showed that our model’s AUC was slightly higher than that of DLpTCR, reaching 0.95. This experiment suggests that our model has the potential to be applied to other antigen peptides derived from other sources and demonstrates its generalizability.

Although iTCep has shown significant improvements in performance metrics, several challenges remain in the area of immunogenicity prediction. Due to the high cross-reactivity of TCR interactions, a single TCR might have the ability to bind to thousands of peptides (Schaap-Johansen et al., 2021). The complexity of the antigen-specific mechanism and the lack of a true negative dataset for TCR-epitope interactions still remain major impediments to the development of methods for predicting unseen-epitopes (Montemurro et al., 2021; Moris et al., 2021). Hence, there is an urgent need to develop cost-effective and accurate computational methods for predicting neoantigen specific recognition by TCR. The model we presented could be regarded as a filter that help researchers to generate a more customized list of potential TCRs or immunogenic peptides for TCR-T engineering and vaccination treatments. The expansion of peptide-TCR binding prediction to consider additional information including V(D)J gene families, TCR CDR3 of α-chain, and epitopes presented by MHC II molecules is an intriguing area for future research. Additionally, molecular biology studies have highlighted the importance of structural and physicochemical homology in TCR cross-reactivity (Milighetti et al., 2021), which will be incorporated into current neoantigen identification pipelines to make a further improvement in our future work.

## Data Availability

Publicly available datasets were analyzed in this study. This data can be found here: http://biostatistics.online/iTCep/#/download.
