# Supplementary material for: iTCep: a deep learning framework for identification of T cell epitopes by harnessing fusion features
Source: Front Genet. 2023 May 9;14:1141535. doi: 10.3389/fgene.2023.1141535 (PMC10203616; doi:10.3389/fgene.2023.1141535)
Supplement: Supplementary file 2 [file Image1.pdf]

Supplementary Material

Article Title

Yu Zhang, Xingxing Jian, Linfeng Xu, Jingjing Zhao, Manman Lu, Yong Lin*, Lu Xie*

*** Correspondence:** Lu Xie (xielu@sibpt.com); Yong Lin (yong_lynn@163.com)

1 Supplementary Figures

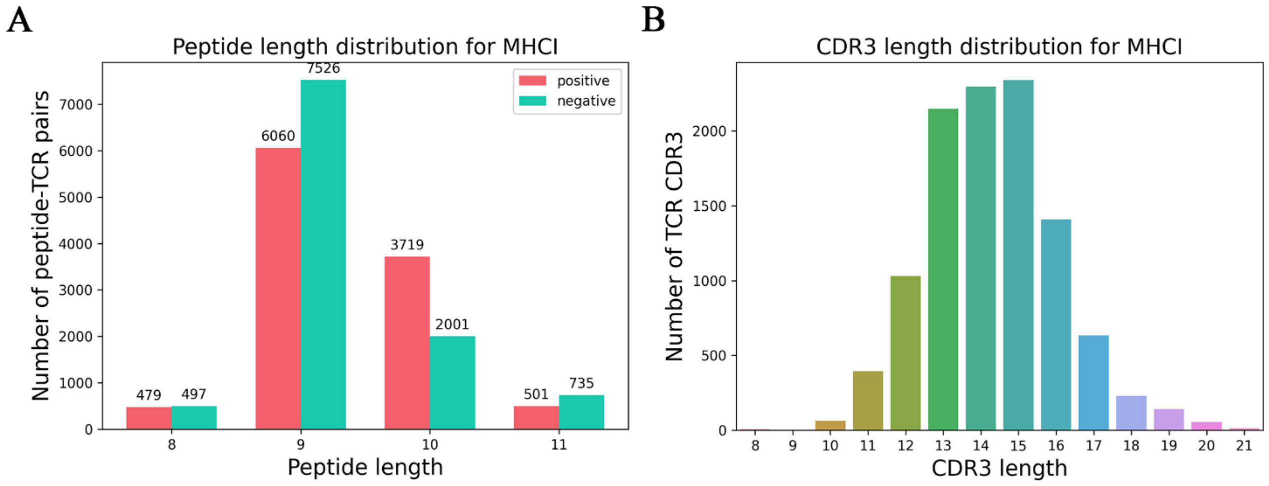

Supplementary Figure 1. Overview of distribution of the filtered dataset. (**a**) Distribution of the number of positive and negative data corresponding to each length of peptide. (**b**) The frequency statistic of the CDR3β sequences in length. The lengths of the 10,759 CDR3 sequences collected from the McPAS-TCR, VDJdb, and IDEB databases range from 8 to 21 amino acids, with the majority falling between 13 and 16.

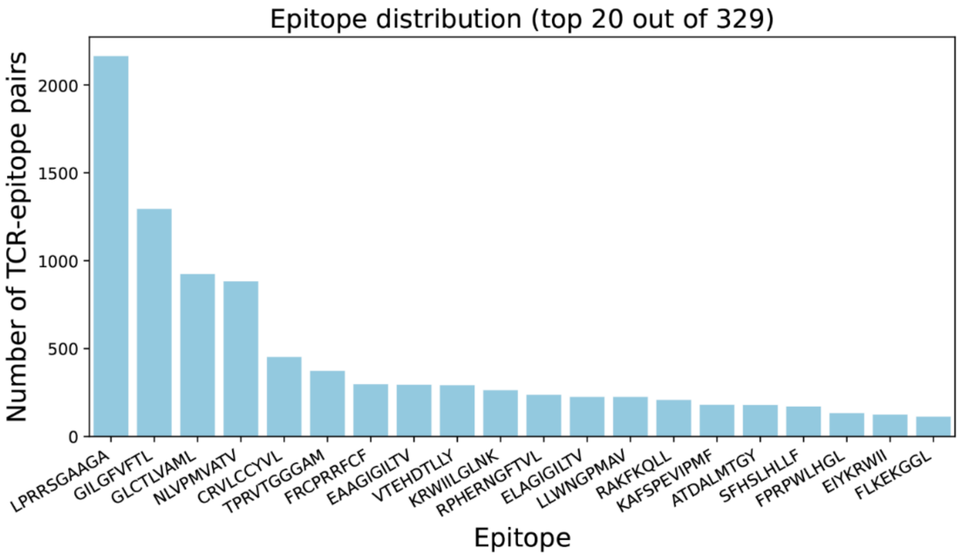

Supplementary Figure 2. The top 20 peptides with the most abundant TCRs out of the overall epitopes.

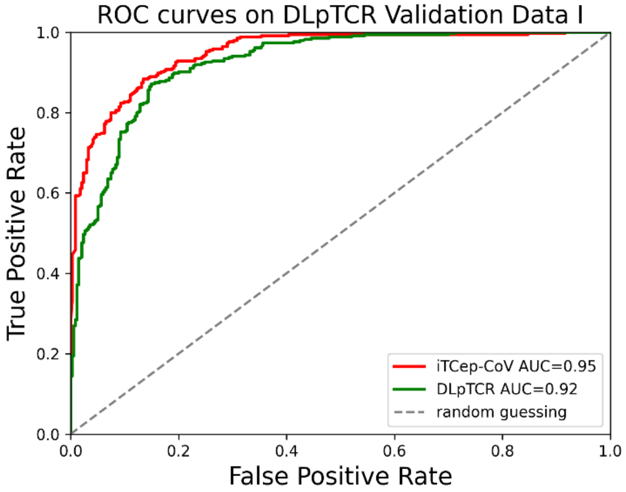

Supplementary Figure 3. Comparison of performance between the transfer learning model of iTCep (iTCep-CoV) and DLpTCR on SARS-CoV-2 epitope and TCR data. The iTCep-CoV model was obtained by transfer learning of the pre-trained iTCep model on COVID-19 data using the following steps: First, we kept all the layers before the fully connected layer of the pre-trained model and added a new output layer to construct a transfer learning model that has one extra fully connected layer compared to the pre-trained model. Then, we froze all the layers from the pre-trained model, compiled, and trained the new model.
